# The Predictive Value of the NEO-FFI Items: Parsing the Nature of Social Anhedonia Using the Revised Social Anhedonia Scale and the ACIPS

**DOI:** 10.3389/fpsyg.2017.00147

**Published:** 2017-02-07

**Authors:** Diane C. Gooding, Emily R. Padrutt, Madeline J. Pflum

**Affiliations:** ^1^Department of Psychology, University of Wisconsin–Madison, MadisonWI, USA; ^2^Department of Psychiatry, University of Wisconsin–Madison, MadisonWI, USA

**Keywords:** social anhedonia, ACIPS, sociability, positive affect, asociality, approach orientation, NEO-FFI

## Abstract

The goal was to examine the nature of social anhedonia using two validated measures and study their relationship to scores on the NEO-Five Factor Inventory (NEO-FFI). Nearly 1,900 college-aged participants completed the Chapman Revised Social Anhedonia Scale (RSAS), Anticipatory and Consummatory Interpersonal Pleasure Scale (ACIPS), and the NEO-FFI. Although both the RSAS and ACIPS were associated with the NEO-FFI domains of Extraversion, Agreeableness, and Neuroticism, linear regression analyses revealed that the RSAS and ACIPS were differentially predicted by NEO-FFI item clusters. The RSAS scores were predicted by Sociability, Nonantagonistic Orientation, Positive Affect, and Activity item clusters. The ACIPS scores were predicted by Sociability, Prosocial Orientation, Activity, and Positive Affect item clusters in addition to gender. In summary, it appears that social anhedonia is multidimensional, associated with various personality domains encompassing social approach and withdrawal.

## Introduction

Social anhedonia is defined as diminished ability to experience pleasure (or a sense or reward) in the interpersonal domain. From an individual differences perspective, social anhedonia can be conceptualized as being on the other end of the continuum from the construct of social connectedness, i.e., individuals possessing social anhedonia experience less reward from interacting with, and/or forming and maintaining relationships with others. From a psychopathology perspective, social anhedonia may be a state- or trait-related symptom, that is, typically associated with less adaptive functioning. As a state-related symptom social anhedonia accompanies disorders, such as major depression and drug withdrawal (Blanchard et al., 2001; Pelizza and Ferrari, 2009; Martinotti et al., 2012). As a trait-related characteristic, it is hypothesized to be indicative of a hypothesized underlying heightened liability for schizophrenia-spectrum disorders (Meehl, 1962; Lenzenweger, 2010). There is increasing evidence supporting the notion that individuals at risk for the later development of schizophrenia and schizophrenia-spectrum disorders are characterized by reduced social and interpersonal pleasure (Kwapil, 1998; Davidson et al., 1999; Gooding et al., 2005, 2007; Miettunen et al., 2011). Regardless of whether social anhedonia is a state- or trait-related in a given individual, it may be an impediment in their progress toward recovery, in terms of seeking and maintaining social support, engaging in group activities (including group therapy) and achieving community integration. One of the aims of research in this area is to further explore the nature of social anhedonia with the eventual goal of remediation.

Social anhedonia is a complex construct, whose exact nature has not yet been parsed. The most well-known direct measure of social anhedonia is the Revised Social Anhedonia Scale (RSAS; Eckblad et al., 1982). A series of studies (Cicero et al., 2015) revealed that variance in the RSAS is accounted for by two factors, namely, Social Apathy/Aversion, and Social Withdrawal. Another measure that is frequently used is the Anticipatory and Consummatory Interpersonal Pleasure Scale (ACIPS; Gooding and Pflum, 2014b). The ACIPS was specifically designed to assess individual differences in the ability to experience pleasure from social and interpersonal interactions, such as sharing experiences and feelings and communicating feelings with others, whether in person or remotely. As such, the ACIPS is an indirect measure of social anhedonia. Research (Gooding and Pflum, 2014a,b) indicates that variance in the ACIPS (Gooding and Pflum, 2014b) is accounted for by four interrelated factors, namely, general social interactions, close relationships, bonding over shared interests and experiences, and family-related interactions. Prior research (Gooding and Pflum, 2014b; Gooding et al., 2015) also indicates that the RSAS and ACIPS are strongly and negatively correlated with each other, though they also have unique aspects of variance.

Investigators have studied the relationship between affect and social anhedonia in nonclinical populations. Socially anhedonic college students (Gooding et al., 2002; Gooding and Tallent, 2003; Horan et al., 2007; Gooding and Pflum, 2011; Pflum et al., 2013) and community-drawn socially anhedonic individuals (Blanchard et al., 2011) display significantly elevated trait negative affect and decreased positive affect (Gooding et al., 2002; Gooding and Tallent, 2003; Kerns et al., 2008; Leung et al., 2010; Blanchard et al., 2011). Gooding and Tallent (2003) observed that socially anhedonic individuals reported significantly greater levels of alexithymia, i.e., difficulty identifying and labeling their feelings, relative to hedonic controls. A study by Martin et al. (2015) extended this research, suggesting that social anhedonia may be uniquely related to alexithymia as well as to decreased trait positive emotion.

Kwapil et al.’s series of Experience Sampling Method (ESM) studies yielded remarkably consistent findings. These investigations were based on nonclinical samples of relatively high-functioning students, and social anhedonia was operational defined by elevated scores on the RSAS. First, social anhedonia was associated with reported diminished positive affect in daily life (Brown et al., 2007; Kwapil et al., 2009, 2012). Socially anhedonic people may feel asocial when in a social situation. The participants with social anhedonia reported experiencing less negative affect when alone (Kwapil et al., 2009). Social anhedonia was associated with a preference for being alone, greater likelihood of being alone, and less enjoyment of social interactions. Furthermore, when participants with social anhedonia were with others, they tended to be in larger and less intimate groups (Kwapil et al., 2009). However, the ESM studies were unable to inform us whether socially anhedonic individuals might contribute in some way to create a non-rewarding social environment. In a study by Llerena et al. (2012), individuals with social anhedonia reported less affiliative affect during a novel social affiliative interaction task. Thus, findings from self-report, behavioral, and ESM methodology provide converging evidence that social anhedonia is associated with decreased interest in, and reward from, social interactions.

To date, investigations of the relations between social anhedonia and the Five Factor Model (FFM) of personality dimensions have relied upon the NEO-PI-R (Costa and McCrae, 1992a). Ross et al. (2002) randomly selected 100 men and 100 women from a total sample of 463 undergraduates in order to examine the association between social anhedonia and the NEO-PI-R domain and facet scales. Neuroticism was significantly associated with RSAS scores in women, though not in men. However, the Neuroticism facet of angry hostility was related to social anhedonia in both genders.

Ross et al. (2002) observed that Extraversion was negatively associated with RSAS scores in both genders, as were the facets of warmth, gregariousness, and positive emotions. The Agreeableness domain and trust facet were inversely associated with social anhedonia in both men and women. Ross et al. (2002) also observed that Openness to Experience was inversely associated with RSAS in women, and the Openness facet of feelings was negatively associated with RSAS scores in both men and women. Conscientiousness was not associated with RSAS scores in either men or women in the Ross et al. (2002) sample.

Kwapil et al. yielded findings that were largely consistent with Ross et al. (2002). In their sample, social anhedonia was negatively associated with Extraversion and inversely associated with facets of warmth, gregariousness, positive emotions, and excitement seeking (Kwapil et al., 2008; Silvia and Kwapil, 2011). Social anhedonia was associated with decreased agreeableness and negatively associated with openness to experience (Kwapil et al., 2008). Silvia and Kwapil (2011) asserted that social anhedonia is characterized by diminished positive affect and decreased interest in social contact, though not increased negative affect.

However, these past studies have not fully exploited the relationship between personality and psychopathology. Moreover, previous attempts to examine the relationship between higher order personality traits and symptoms of mental illness may overlook more specific associations between psychopathology and lower-order personality traits, such as those at the “facet” or “item cluster” level (Klein et al., 2011). An example of the advantages of this approach can be found in a relatively recent study conducted by Spinhoven et al. (2014), who explored the role of various facets of extraversion, namely, positive affectivity, sociability, and activity, in the severity of depressive symptomatology and social avoidance over time. Increasingly, investigators are discovering that differentiating diagnoses and/or symptom profiles at successive levels of a personality hierarchy is possible (Uliaszek et al., 2015). We thought that such an approach, i.e., looking at lower-order personality traits as well as the “big five,” might be useful in terms of taking more of a transdiagnostic approach to understanding social anhedonia.

The goal of the present study was to parse the nature of social anhedonia by measuring it both directly, using the RSAS, and indirectly, using the ACIPS, and investigating the association between those measures and the NEO-Five Factor Inventory (NEO-FFI). The RSAS and ACIPS differ in terms of the directionality and wording of the items; social anhedonia appears to be a multidimensional construct, which may, in turn be differentially related to different personality traits, depending upon how it is assessed. It is noteworthy that the RSAS consists of dichotomous items that have a low rate of endorsement in the general population, whereas the ACIPS contains items that are rated on a Likert-based scale. Thus, it may be possible that the RSAS assesses a more extreme form of social anhedonia. However, by including the full range of scores from both measures, rather than using only the top decile of scores, we included the full continuum of possible responses. This is important because in the past studies, social anhedonia has been investigated primarily from an extreme-groups perspective; the advantage of studying the influence of personality traits is that it affords more of an individual differences perspective.

Based on past findings (Gooding and Pflum, 2014b; Gooding et al., 2015), we expected that the RSAS and ACIPS would be significantly but negatively related to each other. Based on findings by Ross et al. (2002) and Kwapil et al. (2008), we expected that there would be significant relationships between scores on the RSAS and scores on the NEO-FFI domains of Neuroticism, Extraversion, and Agreeableness. We expected nonsignificant or minimal associations between the RSAS and Openness to Experiences and Conscientiousness.

There have been no previous investigations of the ACIPS and any version of the NEO Personality Inventory. Prior research (Gooding et al., 2015) using community adults demonstrated moderately strong associations between total ACIPS scores and scores on scales measuring social connectedness and the need to belong. Thus, we expected to see significant positive associations between total ACIPS scores and scores on the NEO-FFI scales measuring Extraversion and Agreeableness, both primarily dimensions of interpersonal behavior.

Given prior findings (Gooding et al., 2016) indicating negative associations between total ACIPS scores and scores on the Beck Depression Inventory–II (Beck et al., 1996), as well as associations between total ACIPS scores and negative affect (Gooding and Pflum, 2014b) in nonclinical student samples, we expected to see significant inverse associations between total ACIPS scores and scores on the NEO-FFI scale measuring Neuroticism. We did not have directional predictions regarding the Openness to Experience or Conscientiousness domains. We expected that use of Saucier’s (1998) item clusters would provide better delineation of the personality attributes associated with each of the five NEO-FFI domains. We expected to find that the RSAS and ACIPS would be differentially related to, and predicted by, the NEO-FFI item clusters.

## Materials and Methods

### Participants

This was a nonclinical sample drawn from English-speaking undergraduates at a large Midwestern university who were enrolled in introductory Psychology classes during two consecutive semesters. Nearly 2,000 undergraduate students in introductory Psychology classes were recruited to participate in the Mass Survey testing. Inclusion criteria were as follows: undergraduate student status in introductory Psychology classes during those particular semesters; normal or corrected-to-normal vision; and age at least 18 years on the day of assessment. Exclusion criteria included English as a second language and age under than 18 on the day of assessment.

### Measures

We administered the following measures: four of the Chapman psychosis-proneness scales, namely, the Perceptual Aberration (Chapman et al., 1978), Magical Ideation (Eckblad et al., 1983), revised Social Anhedonia (RSAS; Eckblad et al., 1982), and Revised Physical Anhedonia Scale (RPAS; Chapman et al., 1976); the ACIPS (Gooding and Pflum, 2014b); and the NEO Five-Factor Inventory (NEO FFI; Costa and McCrae, 1992b).

#### The Chapman Psychosis-Proneness Scales

All the items from the four Chapman psychosis-proneness scales were randomly mixed together along with Infrequency Scale items and presented in a single questionnaire, the “Survey of Attitudes and Experiences.” Thirteen items from the Chapman Infrequency Scale (Chapman and Chapman, 1983) were included in order to rule out random responding. The resultant questionnaire consisted of 179 true-false items. The Perceptual Aberration scale (Chapman et al., 1978) is a 35-item scale that taps transient body image and perceptual distortions, whereas the 30-item Magical Ideation scale (Eckblad et al., 1983) assesses belief in causality that is invalid. In the present sample, the Perceptual Aberration scale and the Magical Ideation scale showed good internal consistency (α = 0.846 and 0.831, respectively). These scales, which typically correlate highly with each other, are thought to tap positive schizotypy; high scores indicate greater deviance. The Perceptual Aberration Scale and Magical Ideation Scale were therefore administered in order to control for the effect of positive schizotypy, and to provide reference information for future research.

The 61-item Revised Physical Anhedonia scale (RPAS) and 40-item RSAS assess negative schizotypy and are moderately correlated (0.40) with each other. The RPAS measures participants’ inability to experience physical gratification from typically enjoyable stimuli, whereas the RSAS measures schizoid indifference and decreased pleasure in the social domain. In the present sample, the reliability of the RPAS (α = 0.810) and the RSAS (α = 0.855) were also good. Higher scores on each of the Chapman anhedonia scales are associated with greater degrees of anhedonia. The RPAS is typically administered along with the RSAS in order to provide a comparative measure of anhedonia for nonsocial stimuli. This may be particularly informative for some disorders that are characterized by social anhedonia but not physical anhedonia. Although the RSAS is multidimensional in nature, it is traditionally analyzed using the total score.

#### Anticipatory and Consummatory Interpersonal Pleasure Scale (ACIPS)

The 17-item ACIPS (Gooding and Pflum, 2014b) was specifically designed to measure individual differences in the ability to look forward to interactions with others as well as experience pleasure during social/interpersonal interactions as they occurred. The ACIPS is scored on a Likert scale, from 1 (very false for me) to 6 (very true for me), with total scores calculated by summing the ratings (after reversing one negatively-worded item). Although the ACIPS is multidimensional in nature, investigators typically rely upon the total score. Lower scores indicate greater social anhedonia. The psychometric properties of the ACIPS have been discussed elsewhere (see, for example, Gooding and Pflum, 2016). Briefly, the ACIPS has temporal stability, good internal consistency, and demonstrated convergent validity. In the present sample, the internal consistency of the ACIPS was high, ordinal α = 0.958.

#### NEO-Five Factor Inventory (NEO-FFI)

We used the 60-item short form of the NEO Personality Inventory-Revised (NEO-PI-R), the NEO-FFI (Costa and McCrae, 1992b) in order to assess individual differences in personality factors. Based upon a subset of the NEO-PI-R items, the NEO-FFI provides a concise measure of the five basic personality factors, with 12 items for each factor. Each of the items is measured on a Likert-based scale ranging from 0 (“Strongly Disagree”) to 4 (“Strongly Agree”). Nearly half (28 of 60) of the items are reverse-worded. The 13 item-cluster subcomponents are grouped within the five personality domains. In the present sample, the internal consistency of the NEO-FFI was high across the five domains (ordinal α’s ranged from 0.866 to 0.918).

The NEO-FFI does not have the facets contained in the NEO-PI-R. Saucier (1998) derived 13 item clusters for the NEO-FFI domains, which, though not isomorphic with the NEO-PI-R facet scales, correspond generally well with several of them. The 13 factor-analytic derived item clusters and their adjective descriptors provided by Saucier (1997, 1998) provide greater fidelity with which to describe the personality characteristics of the respondents for the NEO-FFI. Readers are referred to Chapman (2007) for further information about Saucier’s item cluster components.

### Statistical Analyses

The data preparation and analyses were performed using SPSS version 23. We computed Cronbach’s alpha coefficients (Cronbach, 1951) to determine the internal consistency of measures scored on a dichotomous scale (e.g., true-false), such as the Chapman psychosis-proneness scales. Ordinal alphas (Zumbo et al., 2007) were computed to determine the internal consistency of measures that were scored on a Likert-type scale, namely, the ACIPS and the NEO-FFI. Pearson product moment correlation analyses were conducted to calculate the association between the questionnaire measures. In order to examine the data for gender differences, independent-samples *t*-tests were performed on mean ACIPS scores, and mean NEO-FFI. All *p*-values are two-tailed; a more stringent level of significance was set at *p* < 0.01. We used Meng’s test (Meng et al., 1992) in order to compare the strength of correlated correlation coefficients. In order to reveal the independent contributions to the prediction of the RSAS or ACIPS, we entered those variables with Pearson correlations at 0.30 or higher with the criterion variable.

### Procedure

Participants were administered a packet of questionnaires, including the measures in the present study, in a large group format. In order to ensure the maximum yield of completed surveys, the surveys relevant to the present study were always at the front of the packet and administered in fixed order. The total administration time was 25 min. This study was approved by the Educational and Social and Behavioral Sciences Institutional Review Board of our institution. All participants provided written informed consent in which they agreed to participate in the study in exchange for extra credit points.

## Results

### Descriptive Statistics for the Sample

A total of 2,026 participants were administered questionnaires. After screening for random responding, incomplete questionnaires, and individuals who did not meet age criteria, the resultant sample consisted of 1890 (876 male, 1,014 female) participants. Demographic characteristics of the sample are summarized in **Table 1**. Briefly, the mean age of the participants was 18.58 (±1.35). It was a predominantly (66.7%) Caucasian sample, reflective of the overall University population. **Table 2** provides the mean scores along with standard deviations for all the self-report measures included in this investigation.

**Table 1:** Demographic characteristics of study sample.

Variable	Total sample	Semester one	Semester two
Total *N*	1,890	1,028	86.2
% Male	46.3	54.0	37.2
Age (years)^a^	18.58 (±1.35)	18.94 (±1.43)	18.22 (±1.26)
Age range	(18–37)	(18–35)	(18–37)
Ethnicity^b^			
White/Caucasian	66.67	63.81	70.07
Black/African American	2.33	1.95	2.78
Asian/Asian American	13.33	11.09	16.00
Latino	2.54	3.11	1.86
Other (not listed)	1.90	2.91	0.70
Multi-ethnic	4.34	5.84	2.55
Did not answer	8.57	11.28	6.03

*Description of the sample in terms of demographic variables of gender, age, and ethnicity. ^a^Age is provided in mean ± SD years; ^b^ethnicity is provided in percent.*

**Table 2:** Descriptive statistics for self-report measures.

	Total sample		Males	Females			
Variable	*M* ±*SD*	α	*M* ±*SD*	*M* ±*SD*	*t*	*p*	*d*
NEO-FFI^a^							
Neuroticism	24.43 ± 6.70	0.88	23.37 ± 6.79	25.34 ± 6.48	6.44	^∗∗∗^	0.30
Extraversion	30.40 ± 6.60	0.92	30.23 ± 6.76	30.55 ± 6.46	0.30	n.s.	
Openness	24.08 ± 5.40	0.90	24.63 ± 5.34	23.61 ± 5.41	4.11	^∗∗∗^	0.19
Agreeableness	32.07 ± 5.96	0.87	30.88 ± 5.98	33.10 ± 5.75	8.20	^∗∗∗^	0.38
Conscientiousness	34.77 ± 6.22	0.87	34.00 ± 6.24	35.43 ± 6.12	5.02	^∗∗∗^	0.23
ACIPS^b^	88.26 ± 8.97	0.96	85.78 ± 9.12	90.40 ± 8.27	11.47	^∗∗∗^	0.53
Chapman scales^c^							
PA	6.25 ± 4.84	0.85	6.34 ± 4.75	6.17 ± 4.91	0.77	n.s.	
MI	9.88 ± 5.53	0.83	9.81 ± 5.52	9.94 ± 5.54	0.49	n.s.	
RSAS	8.51 ± 5.91	0.86	8.90 ± 5.97	8.18 ± 5.85	2.66	^∗∗^	0.12
RPAS	12.26 ± 6.31	0.81	13.84 ± 6.43	10.90 ± 5.87	10.31	^∗∗∗^	0.48

*Descriptive statistics for the male and female participants in terms of the NEO-FFI personality domains, the Anticipatory and Consummatory Interpersonal Pleasure Scale (ACIPS), and Chapman psychosis-proneness scales. ^a^NEO-FFI domains;^b^ACIPS total scores; ^c^Chapman psychosis-proneness scales: PA, Perceptual Aberration; MI, Magical Ideation; RSAS, Revised Social Anhedonia Scale; RPAS, revised Physical Anhedonia Scale. Effect size (Cohen’s *d*) provided for significant group differences.*

*^∗∗^*p* < 0.01 and ^∗∗∗^*p* < 0.001.*

**Table 2** also includes a comparison of the male and female participants in terms of the NEO-FFI factors, ACIPS total scores, and Chapman scale scores. There were sex differences in terms of the NEO personality factors, whereby the female participants reported significantly higher levels of Neuroticism, Agreeableness, and Conscientiousness, and the males reported significantly higher levels of Openness to Experience. Female participants reported significantly higher levels of social and interpersonal pleasure, as measured by the ACIPS, relative to males. Although there were no sex differences in terms of either Perceptual Aberration or Magical Ideation scale scores, the male participants reported significantly higher levels of both social and physical anhedonia.

### Association between the ACIPS and the Chapman Anhedonia Scales

The ACIPS total score was significantly inversely related with the RSAS (*r* = -0.64, *p* < 0.001) and the RPAS (*r* = -0.45, *p* < 0.001) scores. A test of the significance of the difference between two correlation coefficients revealed that the ACIPS was significantly more associated with the RSAS than the RPAS (*Z* = 21.07, *p* < 0.001). Supplementary Table 1 provides a correlation matrix providing the association between the ACIPS and all four Chapman psychosis-proneness scales.

### Associations between the ACIPS and NEO Factors and Clusters

**Table 3** provides the zero-order correlations for the ACIPS total scores and the NEO-FFI domains and clusters. For both males and females, ACIPS total scores were positively associated with Extraversion, Agreeableness, and Conscientiousness, and inversely associated with Neuroticism. In females only, ACIPS total scores were also significantly and inversely associated with Openness to Experience. As indicated in **Table 3**, the total ACIPS score was also associated with several of the item clusters defined by Saucier (1998). **Table 4** provides the predictors of the ACIPS total scores that were revealed from linear multiple regression analyses. In addition to gender, the regression analyses revealed that the Sociability, Prosocial Orientation, Activity, and Positive Affect item clusters significantly predicted the ACIPS scores.

**Table 3:** Zero-order correlations of ACIPS total scores with NEO-FFI domain and clusters.

	ACIPS total scores	
NEO-FFI domain or cluster	Males	Females
	(*n*=876)	(*n*=1,014)
**Neuroticism**	**-0.15^∗∗∗^**	**-0.23^∗∗∗^**
Negative Affect	-0.07^∗^	-0.08^∗^
Self-Reproach	-0.15^∗∗∗^	-0.24^∗∗∗^
**Extraversion**	**0.59^∗∗∗^**	**0.61^∗∗∗^**
Positive Affect	0.46^∗∗∗^	0.46^∗∗∗^
Sociability	0.50^∗∗∗^	0.52^∗∗∗^
Activity	0.45^∗∗∗^	0.46^∗∗∗^
**Openness to Experience**	**-0.01**	**-0.08^∗^**
Aesthetic Interests	0.05	0.03
Intellectual Interests	0.05	0.01
Unconventionality	-0.11^∗∗^	-0.18^∗∗∗^
**Agreeableness**	**0.34^∗∗∗^**	**0.34^∗∗∗^**
Nonantagonistic Orientation	0.22^∗∗∗^	0.26^∗∗∗^
Prosocial Orientation	0.47^∗∗∗^	0.34^∗∗∗^
**Conscientiousness**	**0.24^∗∗∗^**	**0.23^∗∗∗^**
Orderliness	0.11^∗∗^	0.13^∗∗∗^
Goal Striving	0.32^∗∗∗^	0.23^∗∗∗^
Dependability	0.20^∗∗∗^	0.25^∗∗∗^

*Zero-order correlations between the ACIPS total scores and the NEO-FFI personality domains (emboldened) and item accompanying item clusters.*

*^∗^*p* < 0.05, ^∗∗^*p* < 0.01, and ^∗∗∗^*p* < 0.001.*

**Table 4:** Predictors of ACIPS total scores from NEO-FFI item clusters.

Variable	β	95% CI
(Constant)	52.368^∗∗∗^	(50.248, 54.488)
Gender	4.116^∗∗∗^	(3.491, 4.742)
Positive affect	0.446^∗∗∗^	(0.314, 0.578)
Sociability	0.973^∗∗∗^	(0.847, 1.099)
Prosocial orientation	0.698^∗∗∗^	(0.544, 0.852)
Activity	0.660^∗∗∗^	(0.528, 0.793)
*R*^2^	0.434	
*F*	288.42	

*The predictors of the ACIPS (Gooding and Pflum, 2014b) scores from linear multiple regression analyses of the NEO-FFI item clusters. CI, confidence interval. *^∗∗∗^p* < 0.001.*

### Associations between the Chapman Anhedonia Scales and NEO Factors and Item Clusters

**Table 5** provides the zero-order correlations for the RSAS, RPAS, and the NEO-FFI domains and clusters. (Supplementary Table 2 provides the zero-order correlations for the Perceptual Aberration and Magical Ideation scales and the NEO-FFI domains and clusters). As seen in **Table 5**, the RSAS and the RPAS differed in terms of their associations with the NEO domains and item clusters. For both males and females, there were positive associations between Neuroticism and both anhedonia scales. However, the strength of the association was significantly stronger between Neuroticism and social anhedonia; this relationship held true for both males (*Z* = 9.04, *p* < 0.001) and females (*Z* = 11.08, *p* < 0.001).

**Table 5:** Zero-order correlations of psychosis-proneness scores with NEO-FFI domain and clusters.

	Social anhedonia		Physical anhedonia	
NEO-FFI Domain or Cluster	Males	Females	Males	Females
**Neuroticism**	0.30ˆ***	0.39ˆ***	0.14ˆ***	0.11ˆ**
Negative Affect	0.14ˆ***	0.15ˆ***	0.07ˆ*	0.03
Self-Reproach	0.30ˆ***	0.40ˆ***	0.14ˆ***	0.11ˆ**
**Extraversion**	-0.60ˆ***	-0.64ˆ***	-0.35ˆ***	-0.35ˆ***
Positive Affect	-0.49ˆ***	-0.47ˆ***	-0.31ˆ***	-0.30ˆ***
Sociability	-0.57ˆ***	-0.61ˆ***	-0.26ˆ***	-0.24ˆ***
Activity	-0.36ˆ***	-0.41ˆ***	-0.27ˆ***	-0.28ˆ***
**Openness to Experience**	0.05	0.12ˆ***	-0.36ˆ***	-0.30ˆ***
Aesthetic Interests	0.01	0.02	-0.32ˆ***	-0.33ˆ***
Intellectual Interests	0.04	0.10ˆ**	-0.29ˆ***	-0.23ˆ***
Unconventionality	0.05	0.14ˆ***	-0.16ˆ***	-0.08ˆ*
**Agreeableness**	-0.43ˆ***	-0.42ˆ***	-0.18ˆ***	-0.25ˆ***
Nonantagonistic Orientation	-0.35ˆ***	-0.38ˆ***	-0.12ˆ**	-0.21ˆ***
Prosocial Orientation	-0.42ˆ**	-0.31ˆ***	-0.25ˆ***	-0.20ˆ***
**Conscientiousness**	-0.15ˆ***	-0.22ˆ***	-0.09ˆ*	-0.11ˆ**
Orderliness	-0.08ˆ*	-0.17ˆ***	-0.03	-0.09ˆ**
Goal Striving	-0.17ˆ***	-0.19ˆ***	-0.11ˆ**	-0.17ˆ***
Dependability	-0.13ˆ***	-0.21ˆ***	-0.09ˆ**	-0.02

*Zero-order correlations between the RSAS scores (Eckblad et al., 1982), RPAS scores (Chapman et al., 1976) and the NEO-FFI personality domains (emboldened) and accompanying item clusters.*

*^∗^*p* < 0.05, ^∗∗^*p* < 0.01, and ^∗∗∗^*p* < 0.001.*

Similar patterns of associations emerged for Extraversion and Agreeableness. Both males and females displayed significantly negative associations between Extraversion and RSAS scores as well as with RPAS scores. However, the strength of the participants’ association between Extraversion and social anhedonia was significantly greater than their association between Extraversion and physical anhedonia (*Z*’s = 18.47 and 18.23, *p* < 0.001, for males and females, respectively). Similarly, although both males and females displayed inverse associations between Agreeableness and both RSAS and RPAS scores, there was a stronger association with social anhedonia (*Z*’s = 16.49 and 14.78, *p* < 0.001, respectively).

Male participants did not display a relationship between Openness to Experience and their Social Anhedonia scale scores, though the female participants showed a small but positive association. In contrast, both male and female participants revealed a negative association between Openness to Experience and physical anhedonia. Males and females showed inverse correlations between Conscientiousness and Social Anhedonia, as well as between Conscientiousness and Physical Anhedonia; the associations did not differ significantly.

**Table 6** displays the predictors of the RSAS scores obtained from linear multiple regression analyses. The following four NEO item clusters significantly predicted the RSAS scores (Adj. *R*^2^ = 0.482, *p* < 0.001): Sociability, Nonantagonistic Orientation, Positive Affect, and Activity.

**Table 6:** Predictors of RSAS scores.

Variable	β	95% CI
(Constant)	29.354^∗∗∗^	(28.205, 30.503)
Positive affect	-0.316^∗∗∗^	(-0.398, -0.233)
Sociability	-0.921^∗∗∗^	(-1.000, -0.841)
Nonantagonistic orientation	-0.329^∗∗∗^	(-0.374, -0.284)
Activity	-0.310^∗∗∗^	(-0.394, -0.225)
*R*^2^	0.482	
*F*	436.47	

*The predictors of the RSAS (Eckblad et al., 1982) scores from linear multiple regression analyses of the NEO-FFI item clusters. CI, confidence interval.*

*^∗∗∗^p < 0.001.*

## Discussion

The purpose of this investigation was to examine the nature of social anhedonia by measuring it two different ways and investigating the association between those measures with the NEO-FFI. Thus we used the RSAS, which provides a direct measure of social anhedonia, and the ACIPS, which provides an indirect measure. Similar to earlier findings (Gooding and Pflum, 2014b; Gooding et al., 2015), the RSAS scores were significantly and negatively related to total ACIPS scores in both males and females. The strong associations indicated that the measures were assessing related constructs, though there were nonshared sources of variance as well.

Consistent with our predictions, we observed that significant relationships between RSAS scores and scores on the NEO-FFI domains of Neuroticism, Extraversion, and Agreeableness. Overall, these results are consistent with previous findings by Ross et al. (2002) who also observed inverse and significant associations between RSAS and NEO Agreeableness as well as Extraversion. In contrast to Ross et al. (2002) and Kwapil et al. (2008), we found significant associations between the RSAS and NEO Neuroticism in both our male and female participants. Moreover, in contrast to the earlier studies, we observed an inverse and significant correlation between social anhedonia, as measured by the RSAS, and Conscientiousness. One difference between the present study and the earlier investigations is that we relied upon the 60-item NEO-FFI, whereas the other investigations used the 240-item NEO-PI-R, form S. It is noteworthy that the present study includes the largest sample to date in which investigators have looked at the relationship between social anhedonia and personality traits, as measured by the FFM.

We found evidence that the RSAS and ACIPS were measuring different aspects of social anhedonia. Although both the RSAS and ACIPS showed moderate associations with the Neuroticism, Extraversion, and Agreeableness domains of the NEO-FFI, results from the regression analyses indicated that the RSAS and ACIPS were differentially predicted by NEO FFI item clusters. The RSAS scores were predicted by scores on the Sociability, Nonantagonistic orientation, Positive affect, and Activity item clusters. The ACIPS scores were predicted by scores on the Sociability, Prosocial Orientation, Activity, and Positive Affect item clusters in addition to gender. Hedonic capacity for social and interpersonal relationships, as measured by the ACIPS, was predicted by the NEO-FFI’s Prosocial Orientation cluster. However, the NEO-FFI Nonantagonistic orientation cluster was more important in predicting social anhedonia, as measured by the RSAS. The finding that social anhedonia, as measured by higher scores on the RSAS is related to, but distinct from the diminished levels of hedonic capacity for social and interpersonal contact, as measured by the ACIPS, enhances our understanding of the multifaceted nature of social anhedonia. Social anhedonia appears to encompass asociality, affective components, and social withdrawal. Hedonic capacity for social and interpersonal relationships and contact entails sociability, affective components, and social approach.

Given prior findings of increased negative affect among socially anhedonic individuals, we were surprised that negative affect did not predict RSAS scores. Replication of these findings, perhaps with the longer version of the NEO measure, (i.e., the NEO-PI-R) seems prudent.

## Limitations, Strengths, and Future Directions

One limitation of the present investigation is our use of the NEO-FFI rather than the NEO-PI-R. The 30 NEO PI facets are not equally represented by the items on the shortened form of the measure, so it is possible that some of the domains, such as Neuroticism, Extraversion, and Agreeableness, were much better assayed than Openness and Conscientiousness. This line of research would be strengthened by a replication of the present study.

The present investigation is based solely upon self-report ratings. The self-report approach assumes that respondents can accurately reflect upon and report their affective experiences. Another limitation is that we failed to include a measure of alexithymia. Given the association between alexithymia and social anhedonia (Prince and Berenbaum, 1993; Gooding and Tallent, 2003; Martin et al., 2015), it would be useful to examine the extent to which alexithymia might moderate the associations between some of the personality traits and social anhedonia.

The ACIPS is already being used in some intervention research that is focused on the amelioration of negative symptoms (c.f. Nguyen et al., 2016). Further research in the area of social anhedonia appears warranted, perhaps using both measures, along with personality trait assessment, in order to help plan treatments for individuals who present with social anhedonia. Treatment implications may differ for socially anhedonic individuals depending on the personality traits that are most salient. Individuals with social anhedonia, operationally defined by low ACIPS scores, may benefit from cognitive-behavioral treatments that emphasize social skills training (Granholm et al., 2014) and/or experiential therapy (Osimo, 2003; Lilliengren et al., 2016). Individuals with social anhedonia, operationally defined by high RSAS scores, may benefit most from treatments that emphasize behavioral activation training (Dimidjian et al., 2011; Hershenberg et al., 2015), and/or motivational interviewing (Miller and Rollnick, 2013; Romano and Peters, 2016).

Social anhedonia is clearly a transdiagnostic symptom. Another avenue for future research would be to compare the sensitivity of the ACIPS measure to detect social anhedonia in patients with depression, in which the anhedonia may be more state-related or, may reflect an aspect of decisional anhedonia (Treadway and Zald, 2011) and patients with schizophrenia, in which the anhedonia may be more trait-related. Such research would be enhanced by the inclusion of measures of approach and withdrawal as well. Continuing this line of research, and extending it to include the assessment of social anhedonia in people with autism spectrum conditions, and relating measures of the construct to distinct levels of personality traits may assist us in further understanding the ontogeny of psychopathology and disabilities.

In summary, it appears that social anhedonia is multidimensional, associated with various personality domains encompassing social approach and withdrawal.

## Ethics Statement

This study was carried out in accordance with the recommendations of the Educational and Social/Behavioral Institutional Review Board with written informed consent from all subjects. All subjects gave written informed consent in accordance with the Declaration of Helsinki. The protocol was approved by the Educational and Social/Behavioral Institutional Review Board of the University of Wisconsin-Madison.

## Author Contributions

DG is the primary developer of the ACIPS, one of the main measures used in the study. She designed the study, wrote the first draft of the manuscript, analyzed the data, and interpreted the data. EP and MP collected the data and scored the data. EP assisted in interpretation of the data. Both EP and MP assisted in the writing of the manuscript.

## Conflict of Interest Statement

The authors declare that the research was conducted in the absence of any commercial or financial relationships that could be construed as a potential conflict of interest.
