# Supplementary material for: The Predictive Value of the NEO-FFI Items: Parsing the Nature of Social Anhedonia Using the Revised Social Anhedonia Scale and the ACIPS
Source: Front Psychol. 2017 Feb 7;8:147. doi: 10.3389/fpsyg.2017.00147 (PMC5293811; doi:10.3389/fpsyg.2017.00147)
Supplement: Supplementary file 1 [file Table_1.DOCX]

**Supplementary Table 1.**

**Association between the ACIPS and Chapman psychosis-proneness scales.**

Scale 1. 2. 3. 4.

1. ACIPS

2. MI .05*

3. PAB -.08*** .64***

4. RSAS -.64*** .14*** .27***

5. RPAS -.45*** -.13*** -.05* .38***

ACIPS =Anticipatory and Consummatory Interpersonal Pleasure Scale (Gooding & Pflum, 2011, 2014b); MI= Magical Ideation Scale (Eckblad et al., 1983); PAB =Perceptual Aberration Scale (Chapman et al., 1978); RSAS= revised Social Anhedonia Scale (Eckblad et al., 1982); RPAS=revised Physical Anhedonia Scale (Chapman et al., 1976).

*p < 0.05 ** p < 0.01 ***p < 0.001
