# Supplementary material for: The Predictive Value of the NEO-FFI Items: Parsing the Nature of Social Anhedonia Using the Revised Social Anhedonia Scale and the ACIPS
Source: Front Psychol. 2017 Feb 7;8:147. doi: 10.3389/fpsyg.2017.00147 (PMC5293811; doi:10.3389/fpsyg.2017.00147)
Supplement: Supplementary file 2 [file Table_2.DOCX]

**Supplementary Table 2.**

Zero-Order Correlations of Psychosis-Proneness Scores, NEO-FFI Domain and Item Clusters

Perceptual Aberration Magical Ideation

NEO-FFI Domain or Cluster Males Females Males Females

**Neuroticism** .38*** .38*** .32*** .28***

Negative Affect .18*** .11** .13*** .10**

Self-Reproach .39*** .40*** .33*** .29***

**Extraversion** - .08* .11*** -.12*** .04*

Positive Affect -.11** -.12*** .05 -.01

Sociability -.08* -.11*** -.09* .00

Activity .01 - .03 -.16*** .10**

**Openness to Experience** .24*** .30*** .14*** .31***

Aesthetic Interests .26*** .29*** .27*** .35***

Intellectual Interests .17*** .24*** .10** .29***

Unconventionality .08* .11*** -.06 .03

**Agreeableness** -.27*** -.21*** -.20*** -.23**

Nonantagonistic Orientation .-26*** -.25*** -.25*** -.28***

Prosocial Orientation -.17*** -.04 -.01 -.03

**Conscientiousness** - .20*** -.21*** -.03 -.10*

Orderliness -.20*** -.20*** -.09** -.12***

Goal Striving -.09** -.13*** .09** -.04

Dependability -.17*** -.17*** -.03 -.07*

*p < 0.05 **p < 0.01 ***p <0.001
